# A Comparison of Technique Modifications in Laparoscopic Donor Nephrectomy: A Systematic Review and Meta-Analysis

**DOI:** 10.1371/journal.pone.0121131

**Published:** 2015-03-27

**Authors:** Denise M. D. Özdemir-van Brunschot, Giel G. Koning, Kees C. J. H. M. van Laarhoven, Mehmet Ergün, Sharon B. C. E. van Horne, Maroeska M. Rovers, Michiel C. Warlé

**Affiliations:** 1 Department of Surgery, Radboud University Medical Center, Nijmegen, the Netherlands; 2 Department of Health Evidence, Radboud University Medical Center, Nijmegen, the Netherlands; Heinrich-Heine-University and University Hospital Duesseldorf, GERMANY

## Abstract

**Objective:**

To compare the effectiveness of different technique modifications in laparoscopic donor nephrectomy.

**Design:**

Systematic review and meta-analyses.

**Data Sources:**

Searches of PubMed, EMBASE, Web of Science and Central from January 1st 1997 until April 1st 2014.

**Study Design:**

All cohort studies and randomized clinical trials comparing fully laparoscopic donor nephrectomy with modifications of the standard technique including hand-assisted, retroperitoneoscopic and single port techniques, were included.

**Data-Extraction and Analysis:**

The primary outcome measure was the number of complications. Secondary outcome measures included: conversion to open surgery, first warm ischemia time, estimated blood loss, graft function, operation time and length of hospital stay. Each technique modification was compared with standard laparoscopic donor nephrectomy. Data was pooled with a random effects meta-analysis using odds ratios, weighted mean differences and their corresponding 95% confidence intervals. To assess heterogeneity, the I2 statistic was used. First, randomized clinical trials and cohort studies were analyzed separately, when data was comparable, pooled analysis were performed.

**Results:**

31 studies comparing laparoscopic donor nephrectomy with other technique modifications were identified, including 5 randomized clinical trials and 26 cohort studies. Since data of randomized clinical trials and cohort studies were comparable, these data were pooled. There were significantly less complications in the retroperitoneoscopic group as compared to transperitoneal group (OR 0.52, 95%CI 0.33–0.83, I2 = 0%). Hand-assisted techniques showed shorter first warm ischemia and operation times.

**Conclusions:**

Hand-assistance reduces the operation and first warm ischemia times and may improve safety for surgeons with less experience in laparoscopic donor nephrectomy. The retroperitoneoscopic approach was significantly associated with less complications. However, given the, in general, poor to intermediate quality and considerable heterogeneity in the included studies, further high-quality studies are required.

**Trial Registration:**

The review protocol was registered in the PROSPERO database before the start of the review process (CRD number 42013006565).

## Introduction

Living donor nephrectomy is an unusual surgical procedure, since it is performed in healthy individuals. Therefore, it is important that the kidney retrieval procedure is as safe and comfortable as possible, without compromising the function of the graft. In 1954 the first open donor nephrectomy was performed by Murray *et al*. [1]. For many years the technique remained similar, until 1995 when the first laparoscopic donor nephrectomy (LDN) was performed by Lloyd Ratner and Louis Kavoussi [2]. Thereafter several randomized clinical trials have been conducted and showed that LDN was associated with less pain, with equivalent number of complications and perioperative events as compared to the (mini-incision) open technique [3–7]. Although LDN is associated with a longer first warm ischemia time (WIT1), this was not associated with worsening of initial graft function. Based on best available literature nowadays, LDN is considered to be the technique of first choice in western countries [8].

After the introduction of laparoscopic donor nephrectomy, other technique modifications followed: in 1998 the first case of hand-assisted laparoscopic donor nephrectomy (HALDN) was performed [9]. Hand-assistance has not only the theoretical advantage of direct tactile feedback, but also the possibility of manual dissection. The retroperitoneoscopic approach may reduce the risk of injury to the bowel or other visceral organs. Visceral injuries are the second most frequent complications in laparoscopic urological procedures, second to vascular injuries [10]. In 1994 the first retroperitoneoscopic donor nephrectomy was performed and in 2002 the retroperitoneoscopic approach and hand-assistance were combined [11–12].

To date, several modifications of the standard transperitoneal laparoscopic approach have been applied, including: hand-assisted transperitoneal laparoscopic, total retroperitoneoscopic, hand-assisted retroperitoneoscopic, laparoendoscopic single site (LESS) and the natural orifice (NOTES) approach. All technique modifications claim to have specific advantages. Advantages of hand-assistance are the possibility of manual compression in case of bleeding, quicker kidney removal and better spatial orientation, in theory leading to less blood loss (EBL), shorter WIT1 and shorter operation time (ORT) when compared to conventional laparoscopy [13]. The retroperitoneal access theoretically lowers the risk of injuries to intra-abdominal organs [14,15]. Others claim LESS is associated with quicker postoperative recovery, less pain and better cosmetic outcome because the entire incision is hidden in the umbilicus [16,17].

Many studies comparing technique modifications of LDN with the standard laparoscopic approach have been performed, varying from relatively small case series to randomized clinical trials [13,16–18]. To determine which technique is “best”, it is important that all outcome measures that are critical or important for decision making, are carefully balanced from the donors’ perspective. Therefore, a systematic review and meta-analysis (SRMA) is indicated to compare the effectiveness of different technique modifications in laparoscopic donor nephrectomy.

## Methods

A systematic review and meta-analysis (SMRA) was performed in accordance with the PRISMA (Preferred Reporting Items for Systematic reviews and meta-analyses) [19] guidelines and used a predetermined protocol, registered in PROSPERO (www.crd.york.ac.uk/prospero, CRD number 42013006565) [20].

### Search

The Cochrane Central Register of Controlled Trials (CENTRAL) in the Cochrane Library, PubMed, EMBASE and Web of Science were searched from January 1^st^, 1997. The search was performed on March 1^st^ 2013 and was updated on April 1^st^ 2014. The search strategy is provided in Table 1. No limits regarding language, blinding or publication status were used. The references of the identified trials and cross references were searched to identify any further relevant randomized clinical trials or cohort studies. When multiple studies describing the same population were published, the most complete report was used.

**Table 1:** Search strategy.

**Database**	**Search strategy**
PubMed	(“Tissue Donors”[Mesh] OR “living donors”[Mesh] OR ((live[tiab] or living[tiab]) AND donor[tiab]) OR ((live[tiab] OR living[tiab]) AND kidney[tiab]) AND “kidney transplantation”[Mesh] OR “nephrectomy”[Mesh] OR kidney[tiab] OR kidneys[tiab] OR renal[tiab]) AND (“laparoscopy”[Mesh] OR laparoscop*[tiab] OR laparoendosc*[tiab] OR laparo-endoscop*[tiab] OR RALDN[tiab] OR RADN[tiab] OR RALN[tiab] OR HAL[tiab] HALDN[tiab] OR HALN[tiab] OR HLDN[tiab] OR LDN[tiab] OR SPL[tiab] OR HARP[tiab] OR retro-peritoneoscop*[tiab] OR trans-peritoneoscop*[tiab] OR extra-peritoneoscop*[tiab] retroperitoneoscop*[tiab] OR transperitoneoscop*[tiab] OR extraperitoneoscop*[tiab] OR single-port[tiab] OR single port[tiab])
Embase	Living donor OR kidney donor OR ((live:ti,ab OR living:ti,ab) AND (donor:ti,ab OR donors:ti,ab)) OR ((live:ti,ab OR living:ti,ab) AND kidney:ti,ab) OR (kidney transplantation OR nephrectomy OR kidney:ti,ab OR kidneys:ti,ab OR renal:ti,ab) AND (exp* laparoscopy OR laparoscop* OR laparoendoscop* OR laparo-endoscop* OR RALDN OR RADN OR RALN OR HAL OR HALDN OR HALN OR HLDN OR LDN OR SPL OR HARP OR retro-perito* OR transperito* OR extra-perito* OR retroperito* OR transperito* OR extraperito* OR single-port OR single port)
Web of science	TS = (((Live OR living*) AND (donor OR donors)) OR ((live or living*) AND kidney*) AND (nephrect* OR kidney OR kidneys OR renal) AND (laparoscop* OR laparoendosc* OR laparo-endosc* OR RALN OR RADN OR RALN OR HAL OR HALDN OR HLDN OR LDN OR SPL OR HARP OR retro-peritoneoscop* OR trans-peritoneoscop* OR extra-periteonscop* OR retro-peritoneoscop* OR transperitoneoscop* OR extraperitoneoscop* OR single-port OR single port))

[tiab]: word in title or abstract

[mesh]: medical subheading, controlled vocabulary as used by National Library or Medicine for indexing articles

*: truncation; retrieves all possible suffix variations of root word indicated

### Study selection

Two authors (DÖ and ME) independently confirmed the eligibility of studies and collected the data from the qualifying studies. Potentially relevant studies were obtained and the full text was examined. Studies were eligible if the standard transperitoneal laparoscopic technique was compared to one of the technique modifications (*i*.*e*. hand-assisted (HALDN), retroperitoneoscopic (RDN), hand-assisted retroperitoneoscopic (HARDN) or laparoendoscopic donor nephrectomy (LESS) and at least one of the quantitative outcome measures mentioned in the next section of this paper was included. Standard LDN was performed by a transperitoneal approach in the peritoneal cavity, while retroperitoneoscopic donor nephrectomy (RDN) was performed in the retroperitoneal space. Hand-assistance was performed by introduction of the surgeon’s hand in the peritoneal cavity or retroperitoneal space to facilitate the dissection and extraction of the kidney. In LESS the procedure is performed through a single port that was introduced through the umbilicus.

Studies published only as abstracts were excluded, because a thorough quality assessment could not be performed, also case series with less than 10 patients were excluded. Allowed study designs were: randomized clinical trials and comparative prospective or retrospective cohort studies.

### Data extraction

For each included trial the following characteristics were extracted: year of publication, country and city (or cities), single- or multicenter design, study design, total number of patients, total number of patients in each treatment arm, mean age and standard deviation, gender, mean body mass index (BMI) and standard deviation and number of left donor nephrectomies.

The primary outcome measure was the number of complications. Secondary outcome measures include: conversion to open donor nephrectomy (ODN), WIT1 (seconds), EBL (mL), graft function, ORT (minutes) and length of hospital stay (LOS) (days). Graft function was reflected by the incidence of delayed graft function (DGF). DGF was defined as the need for dialysis in the first week after transplantation, excluding dialysis for hyperkalemia [21].

In case of missing data, the corresponding author was addressed for more information. In case the authors did not reply, the standard deviation was reconstructed from the mean and range, if sufficient data was available.

### Risk of bias assessment

For randomized clinical trials a quality assessment was performed according to the Cochrane Risk of Bias Tool [22]. For the non-randomized trials the quality assessment was performed using the adapted Newcastle Ottawa Rating scale, which consists of three factors: patient selection, comparability of study groups and assessment of outcome [23]. In this adapted quality assessment scale a maximum of 7 stars could be scored; 6 or 7 stars corresponded with high quality, 4 or 5 stars with intermediate quality and 0 to 3 stars with low quality. The quality assessments were performed by two authors (DÖ and ME) independently. In case of discrepancies, consensus was reached by discussion.

### Statistical methods

The meta-analysis for randomized clinical trials and cohort studies separately was performed using Review Manager (version 5.2 The Cochrane Collaboration, Oxford, UK). When the mean difference or odds ratio of the randomized clinical trial coincided with the 95% CI of the cohort studies, the data were pooled.

Data was pooled with a random effects meta-analysis with odds ratios, weighted mean differences and their corresponding 95% CIs. To assess heterogeneity in results of individual studies, the I^2^ statistic was used (I^2^ > 75% was used as a threshold indicating significant heterogeneity). Reasons for heterogeneity were explored. Publication bias was assessed with Funnel plots and Egger’s regression model. All analyses were performed on an intention-to-treat bias.

### Sensitivity analysis

For the sensitivity analysis, the poor quality studies were excluded. For each technique modification, the trials were divided in two equal groups based on the year of publication (earliest and latest) and all outcome measures were re-analyzed, *i*.*e*. the results of an initial group were compared to the results of the second (last) group. Also the data was re-analyzed with regard to the number of complications for each technique modification after excluding all mild complications per study. For this, all individual complications were classified according to their estimated severity (*i*.*e*. severe, moderate or mild). Severe complications were defined as (potentially) live-threatening events, (multi-) organ failure or events necessitating admission to the Intensive Care Unit. Events (potentially) requiring an intervention or surgery were defined as moderate complications. Mild complications only required non-surgical or conservative management.

## Results

### Search results

The search strategy identified 509 potential eligible studies, the titles and abstracts were screened for inclusion. The full text of 137 articles was retrieved, of which 31 met the inclusion criteria. Reasons for exclusion of the remaining articles were: not full-text available (*n* = 2), the study group contained less than 10 patients (*n* = 5), irrelevant endpoint (*n* = 8), other urological procedures were also included (*n* = 15) or because LDN was not included in the comparison (*n* = 76).

**Fig 1:**
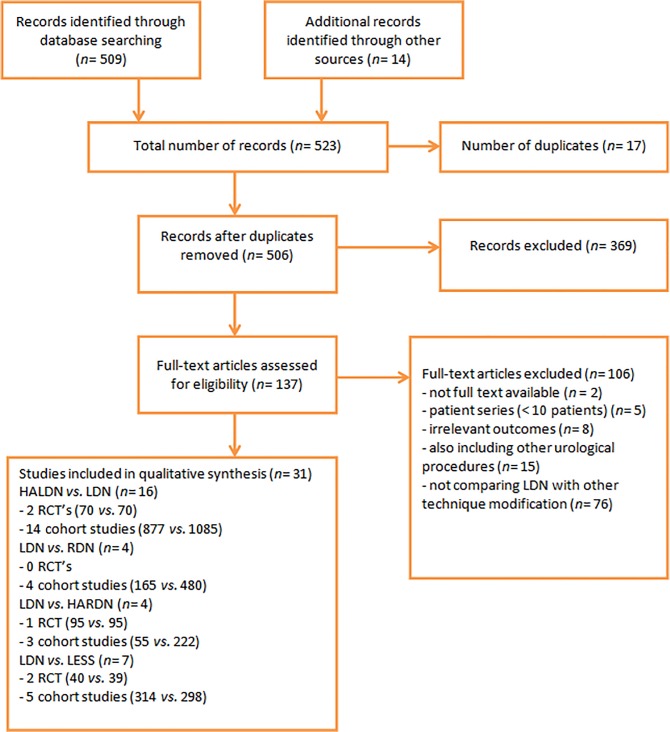
PRISMA flow chart of study search [19].

Fig. 1 shows the characteristics of the 31 included studies. Sixteen studies compared HALDN *versus* LDN, 4 studies compared RDN *versus* LDN, 4 compared HARDN *versus* LDN and 7 studies compared LESS *versus* LDN, study characteristics are shown in Table 2 and 3. 25 Authors were contacted by e-mail for additional information, one author provided additional information. Table 4 and 5 show the risk of bias for all included trials and cohort studies, respectively. The overall quality of the included studies was low to moderate.

**Table 2:** Characteristics of randomized clinical trials.

Author	Year of publication	Country/ State	Comparison*	Number of patients (LDN vs technique modification)
Bargman [18]	2006	US/Indiana	HALDN	20 *vs*. 20
Cho [25]	2013	Korea	HALDN	50 *vs*. 50
Dols [42]	2014	the Netherlands	HARDN	95 *vs*. 95
Kurien [17]	2011	India	LESS	25 *vs*. 25
Richstone [43]	2013	US/New York	LESS	14 *vs*. 15

* *with LDN as reference*

**Table 3:** Characteristics of cohort studies.

**Study**	**Year of publication**	**Country/ State**	**Comparison(s)** *	**Number of patients (technique modifications *versus* LDN)**
Afaneh [16]	2011	US/New York	LESS	50 *vs*. 50
Barth [45]	2013	US/Maryland	LESS	6 *vs*. 6
Branco [24]	2008	Brazil	HALDN	67 *vs*. 89
Canes [46]	2010	US/Vermont	LESS	17 *vs*. 17
El-Galley [26]	2004	United Kingdom	HALDN(ODN)	55 *vs*. 17 (vs 28)
Gao [39]	2007	China	RDN	19 *vs*. 28
Gershbein [27]	2002	US/California	HALDN	15 *vs*. 30
Gjertsen [47]	2006	US/New York	HARDN (ODN)	11 *vs*. 15 (vs 25)
Kocak [28]	2007	US/Ilinois	HALDN	318 *vs*. 482
Lai [29]	2010	Taiwan	HALDN	45 vs 52
Lunsford [44]	2011	US/California	LESS	10 *vs*. 20
Mateo [30]	2003	US/California	HALDN	18 *vs*. 29
Mjoen [31]	2009	Norway	HALDN/HARDN	177 *vs*. 26 *vs*. 196
Ng [40]	2004	US/Cleveland	RDN	36 *vs*. 107
Percegona [32]	2008	Brazil	HALDN	34 *vs*. 21
Ruiz-Deya [33]	2001	US/Louisiana	HALDN	11 *vs*. 23
Ruszat [34]	2006	Switzerland	HALDN/RDN(ODN)	33 *vs*. 63 *vs*. 12 (vs 69)
Salazar [35]	2005	Canada	HALDN(ODN)	15 *vs*. 24 (*vs*. 11)
Srivastava [41]	2008	India	RDN	38 *vs*. 342
Stamatakis [47]	2013	Texas	LESS	102 *vs*. 111
Ungbhakorn [36]	2012	Thailand	HALDN(ODN)	23 *vs*. 82 (*vs*. 95)
Velidedeoglu [37]	2002	US/Pennsylvania	HALDN(ODN)	60 *vs*. 40 (*vs*. 50)
Wadström [38]	2003	Sweden	HALDN/HARDN	14 *vs*. 18 *vs*. 11

* *with LDN as reference*

**Table 4:** Risk of bias assessment for randomized clinical trials.

	**Random sequence generation**	**Allocation concealment**	**Blind of participants and personnel (performance bias)**	**Blinding of outcome assessment (detection bias)**	**Incomplete outcome data (attrition bias)**	**Selective reporting (reporting bias)**	**Funding**	**Other bias**
Bargman [18]	?	?	-	?	+	+	+	None
Cho [25]	-	?	-	+	+	+	+	None
Dols [42]	+	?	+	?	+	+	+	None
Kurien [17]	?	?	?	+	+	+	+	Experience of the surgeon with less technique unclearInclusion of patients with high BMI during the study
Richstone [43]	+	+	+	?	-	+	+	Premature terminationExperience with LESS is unclear

**Table 5:** Quality assessment of cohort studies.

	Comparability			Exposure			Total
	Representativeness	Follow up	Comparability	Ascertainment	Same method	Non response rate	
**HALDN**							
Branco [24]	*		**	*	*		5
El-Galley [26]	*		*	*	*		4
Gershbein [27]	*		**		*		4
Kocak [28]	*		**	*	*		5
Lai [29]	*	*	*	*	*		5
Mateo [30]	*		*	*	*		4
Mjoen [31]	*		*	*	*		4
Percegona [32]	*		*	*	*		4
Ruiz-Deya [33]		*	*	*	*		4
Ruszat [34]	*		*	*	*		4
Salazar [35]	*		*	*	*		4
Ungbhakorn [36]	*		*	*	*		4
Velidedeoglu [37]	*		*	*	*		4
Wadstrom [38]	*		*	*	*		4
**RDN**							
Gao [39]			*		*		2
Ng [40]	*		*	*	*		4
Ruszat [34]	*		*	*	*		4
Srivastava [41]	*		*		*		3
**HARDN**							
Mjoen [31]	*		*	*	*		4
Gjertsen [47]	*		*		*		3
Wadstrom [38]	*		*	*	*		4
**LESS**							
Afaneh [16]	*	*	**	*	*		6
Barth [45]	*		*	*	*	*	5
Canes [46]	*		**	*	*		5
Lunsford [44]	*		*	*	*		4
Stamatakis [47]	*		**	*	*		5

*0–3 low quality*

*4–5 intermediate quality*

*6–7 high quality*

### HALDN versus LDN

**Fig 2:**
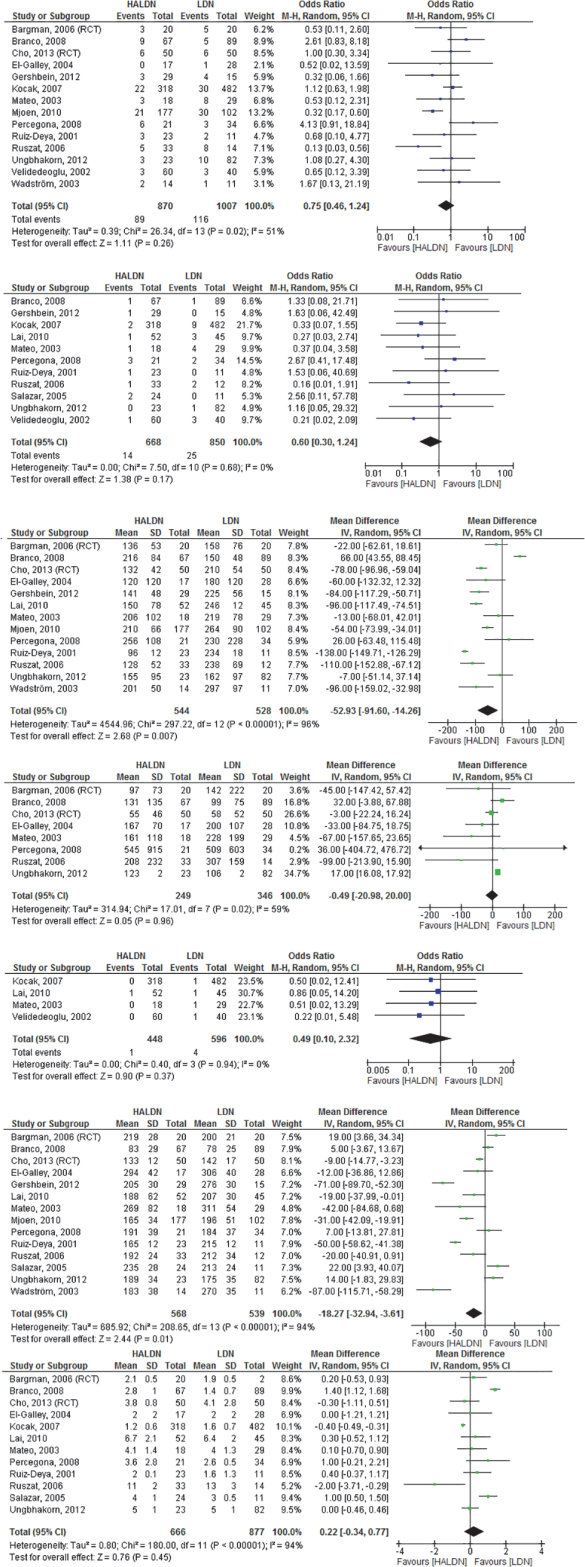
Forrest plots comparing HALDN versus LDN (RCT’s and cohort studies combined). a. Complications in HALDN versus LDN. Fourteen studies described the occurrence of complications in HALDN versus LDN (OR 0.75, 95% CI 0.46–1.24, I^2^ = 51%). b. Conversion to ODN in HALDN versus LDN. Eleven studies described the number of conversions to ODN in HALDN versus LDN (OR 0.61, 95% CI 0.30–1.25, I^2^ = 0%). c. WIT1 in HALDN versus LDN. Thirteen studies described WIT1 in HALDN versus LDN (MD -52.99, 95% CI -91.57- -14.41, I^2^ = 96%). WIT1 was significantly shorter for HALDN. d. EBL in HALDN versus LDN. Eight studies described EBL in HALDN versus LDN (MD -0.49, 95% CI -20.98–20.00, I^2^ = 59%). e. Graft function in HALDN versus LDN. Four studies described the incidence of delayed graft function in HALDN versus LDN (OR 0.49, 95% CI 0.10–2.32, I^2^ = 0%). f. ORT in HALDN versus LDN. Fourteen studies described ORT in HALDN versus LDN (MD -18.27, 95% CI -32.90- -3.64, I^2^ = 94%). ORT was significantly shorter in the HALDN group. f. LOS in HALDN versus LDN. Twelve studies described LOS in HALDN versus LDN (MD 0.22, 95% CI -0.34–0.77, I^2^ = 95%).

Sixteen studies (2 randomized clinical trials and 14 cohort studies) compared HALDN to LDN (Fig. 2) [18,24–38]. No differences were found in the number of complications, conversion to ODN, EBL, graft function and LOS (Fig. 2A, 2B, 2D, 2E and 2G). Graft function was only studied in 4 cohort studies (Fig. 2E). WIT1 and ORT were shorter in HALDN as compared to LDN (MD = -52.9, 95%CI -91.6 - -14.3, I^2^ = 96% and MD = -18.3, 95%CI -32.9 - -3.6, I^2^ = 94%, respectively) (Fig. 2C and 2F).

### RDN versus LDN

**Fig 3:**
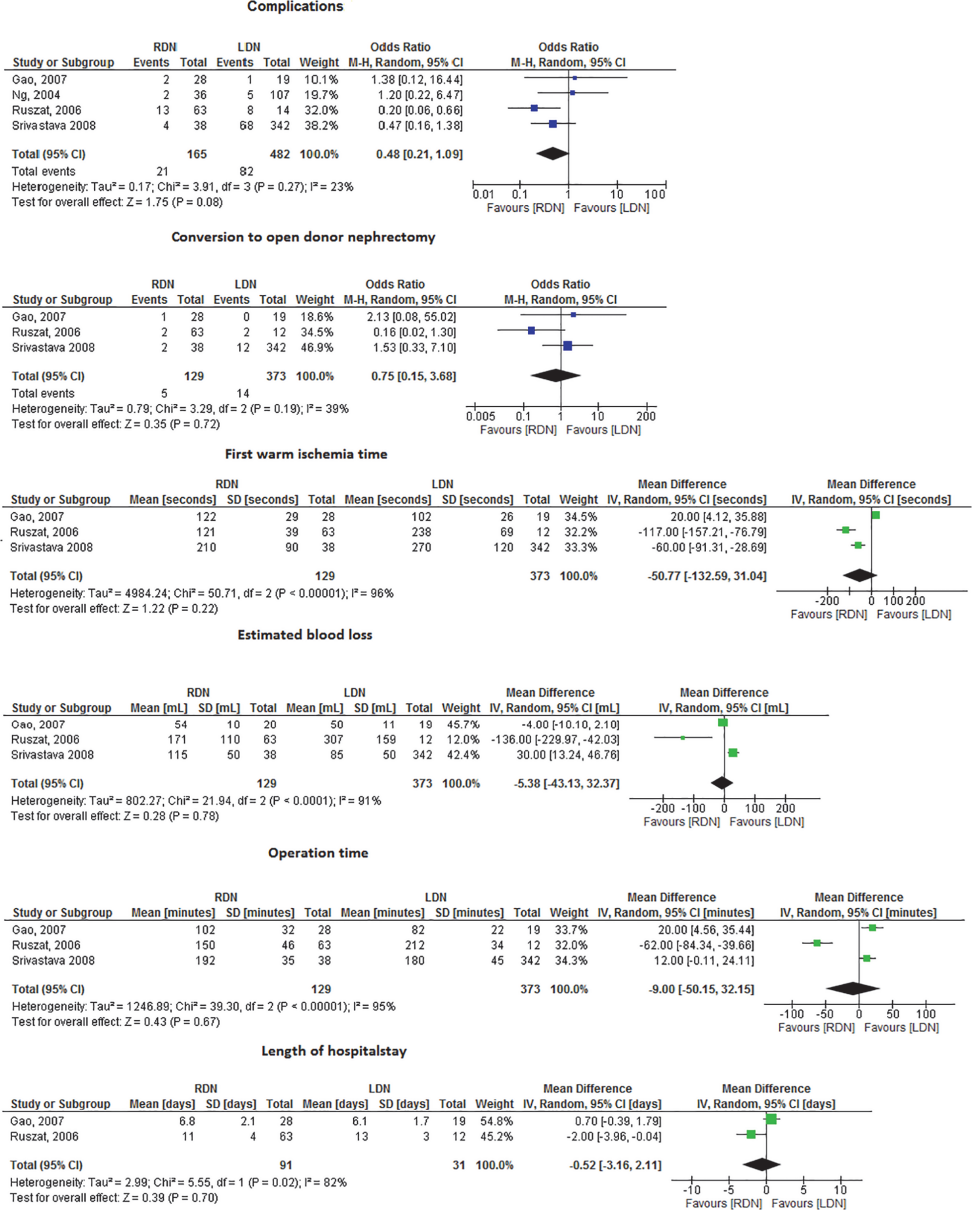
Forrest plots comparing RDN versus LDN (RCT’s and cohort studies combined). a. Complications of RDN versus LDN. Four studies compared complications in RDN versus LDN (OR 0.48, 95% CI 0.21–1.09, I^2^ = 23%, there was a tendency towards less complications for RDN. b. Conversion to ODN in RDN versus LDN. Three studies compared the number of conversions to ODN in RDN versus LDN (OR 0.75, 95% CI 0.15–3.68, I^2^ = 39%). c. WIT1 in RDN versus LDN. Three studies described WIT1 in RDN versus LDN (MD -50.77, 95%CI -132.59–31.04, I^2^ = 96%). d. EBL in RDN versus LDN. Three studies described EBL in RDN versus LDN (MD -5.38, 95% CI -43.13–32.37, I^2^ = 91%0. e. ORT in RDN versus LDN. Three studies described ORT in RDN versus LDN (MD -9.00, 95% CI -50.15–32.15, I^2^ = 95%). f. LOS in RDN versus LDN. Two studies described LOS in RDN versus LDN (MD -0.52, 95% CI -3.16–2.11, I^2^ = 82%).

Four (cohort) studies compared RDN to LDN (Fig. 3) [39–41]. A tendency towards less complications was seen in the RDN group (OR = .5, 95%CI. 2–1.1, I^2^ = 23%) (Fig. 3A). With regard to the intra-abdominal injuries, studies comparing RDN with LDN described 1 splenic and 2 bowel injuries, all occurring in the LDN group. Conversion to ODN, WIT1, EBL, ORT and LOS did not differ between both types of intervention (Fig. 3B, 3C, 3D, 3E and 3F). Graft function could, due to insufficient data, not be analyzed (data not shown).

### HARDN versus LDN

**Fig 4:**
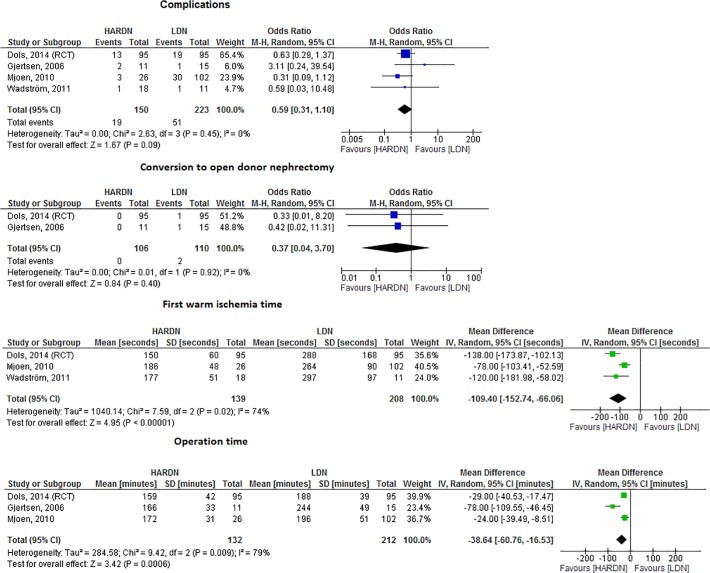
Forrest plots comparing HARDN versus LDN (RCT and cohort studies combined). a. Complications of HARDN versus LDN. Four studies compared complications in HARDN versus LDN (ORT 0.59, 95% CI 0.31–1.10, I^2^ = 0%), there was a tendency towards less complications for HARDN. b. Conversion to ODN in HARDN versus LDN. Two studies compared the number of conversions to ODN in HARDN versus LDN (OR 0.37, 95%CI 0.04–3.70, I^2^ = 0%). c. WIT1 (seconds) in HARDN versus LDN. Three studies described WIT1 in HARDN versus LDN (MD -109.40, 95% CI-152.74- -66.06, I^2^ = 74%). HARDN was associated with shorter WIT1. d. ORT (minutes) in HARDN versus LDN. Three studies described ORT in HARDN versus LDN (MD -38.64, 95% CI -60.76- -16.53, I^2^ = 79%). HARDN was associated with shorter ORT.

Three cohort studies and 1 randomized clinical trial compared HARDN to LDN (Fig. 4) [34,39–42]. A tendency towards less complications was found in the HARDN group when compared to LDN (OR = .6, 95%CI. 3–1.1, I^2^ = 0%) (Fig. 4A), all intra-abdominal injuries occurred in the LDN group (*i*.*e*. 2 splenic and 3 bowel injuries). No differences in the number of conversions to ODN or graft function were found (Fig. 4B). WIT1 and ORT were shorter in HARDN as compared to LDN (MD = -109.4, 95%CI -152.7 - -66.1, I^2^ = 74% and MD = -38.6, 95%CI -60.8- -16.5, I^2^ = 79%, respectively), Fig. 4C and 4D).

### LESS versus LDN

**Fig 5:**
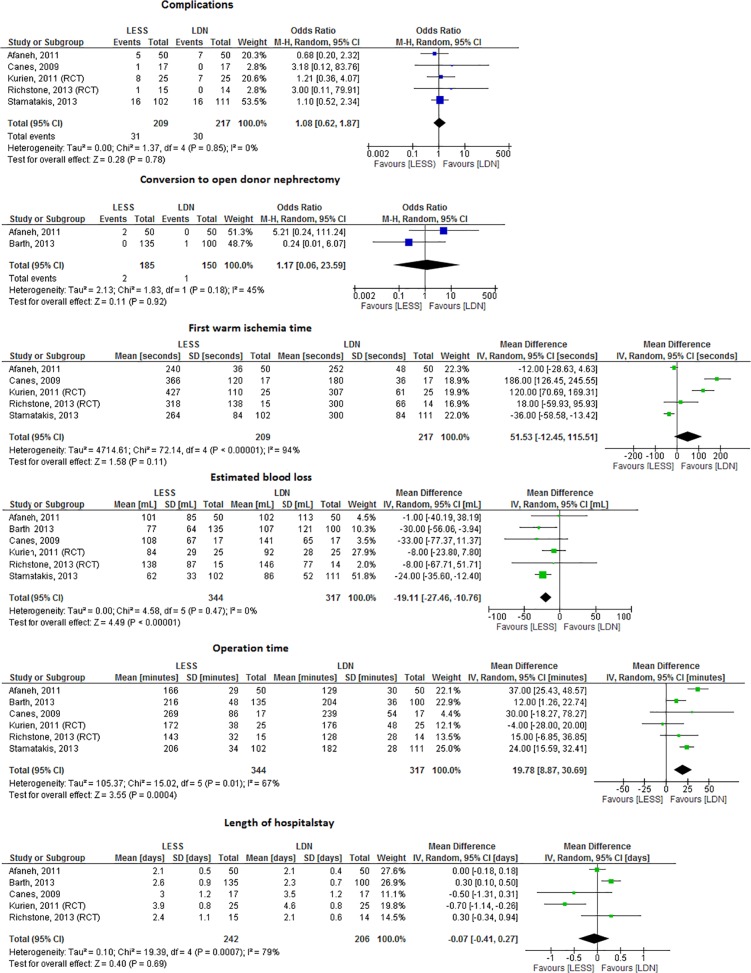
Forrest plots comparing LESS versus LDN (RCT and cohort studies combined). a. Complications of LESS versus LDN. Five studies compared the number of complications in LESS versus LDN (OR 1.08, 95%CI 0.62–1.87, I^2^ = 0%). b. Conversion to ODN in LESS versus LDN. Two studies compared the number of conversion to ODN in LESS versus LDN (OR 1.17, 95%CI 0.06–23.59, I^2^ = 45%). c. WIT1 (seconds) in LESS versus LDN. Five studies described WIT1 in LESS versus LDN (MD 51.53, 95%CI -12.45–115.51, I^2^ = 94%). d. EBL (mL) in LESS versus LDN. Three studies described EBL in LESS versus LDN (MD -19.11, 95%CI 27.46 –-10.76, I^2^ = 0%). LESS was associated with significantly less EBL. e. ORT (minutes) in LESS versus LDN. Six studies compared ORT in LESS versus LDN (MD 19.78, 95% CI 8.87–30.69, I^2^ = 67%). ORT was significantly longer for LESS. f. LOS (days) in LESS versus LDN. Five studies compared LOS in LESS versus LDN (MD -0.07, 95% CI -0.41–0.27, I^2^ = 79%).

Seven studies, 2 randomized clinical trials [17,43] and 5 cohort studies [16,44–47] compared LESS and LDN (Fig. 5). There was no difference in the number of complications, conversions to ODN, WIT1 and LOS (Fig. 5A, 5B, 5C and 5F). Less EBL was seen in LESS as compared to LDN (MD = -19.1, 95%CI -27.5 - -10.8, I^2^ = 0%), while OR time was shorter in LDN as compared to LESS (MD = 19.8, 95%CI 8.9–30.7, I^2^ = 67%) (Fig. 5D and 5E).

### Retroperitoneal versus transperitoneal approach

**Fig 6:**
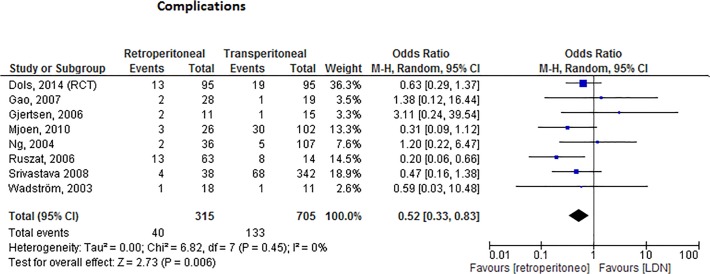
Forest plot comparing the number of complications in the retroperitoneoscopic group (HARDN and RDN) versus the transperitoneal approach. The number of complications was significantly lower in the retroperitoneoscopic group (OR 0.52, 95%CI 0.33–0.83, p<0.01).

When the complications of both retroperitoneal approaches (HARDN and RDN) were combined and compared to the transperitoneal approach, the retroperitoneoscopic approaches were associated with significantly less complications, Fig. 6 (OR 0.52, 95%CI 0.33–0.83, I^2^ = 0%).

### Hand-assisted versus fully laparoscopic approach

**Fig 7:**
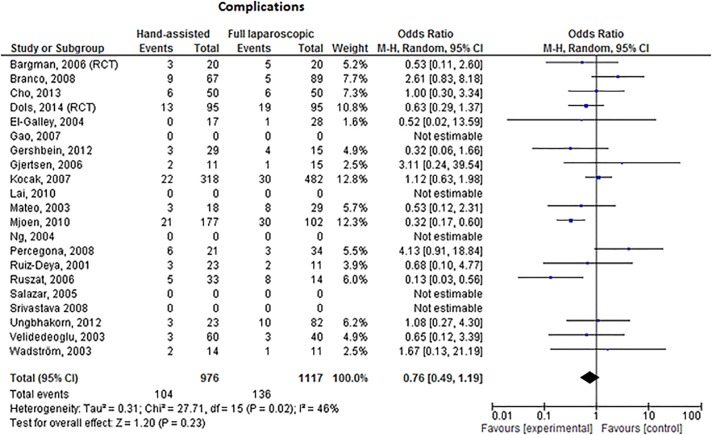
Forest plot comparing the number of complications in the hand-assisted versus the fully laparoscopic donor nephrectomy. No difference in complications was found (OR 0.76, 95% CI 0.49–1.19, p = 0.23).

No significant difference in the number of complications was demonstrated when the hand-assisted techniques (HALDN and HARDN) were combined and compared to the fully laparoscopic technique, Fig. 7 (OR 0.52, 95% CI 0.33–0.83, I^2^ = 46%).

### Sensitivity analysis

For the sensitivity analysis, the poor quality cohort studies (less than 3 points) were excluded [38, 40, 41]. Since 2 of the 3 studies that had to be excluded compared RDN with LDN, which included only 4 studies, insufficient data remained for a meaningful analysis. No significant changes occurred when the third study was excluded from the analysis.

**Fig 8:**
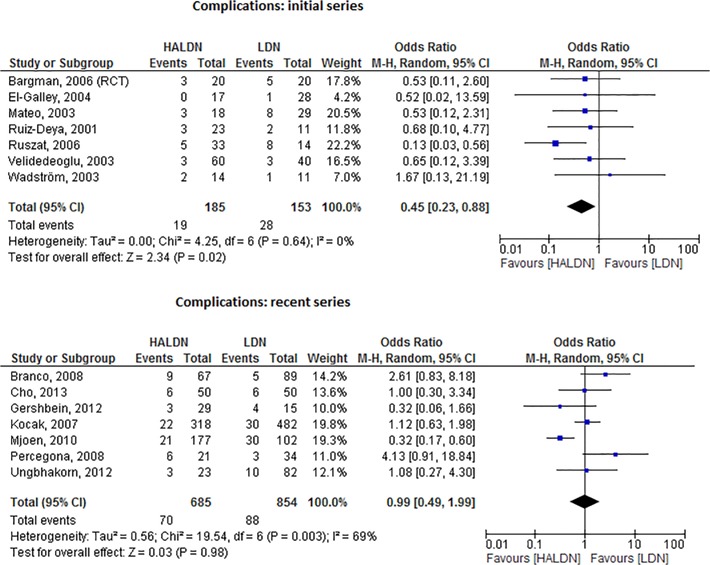
8a: Forest plot comparing initial series of HALDN versus LDN (RCT and cohort studies combined). Six studies were included. The initial series of HALDN were associated with less complications (OR 0.45, 95% CI 0.23–0.88, p = 0.02). 8b: Forest plot comparing recent series of HALDN versus LDN (RCT and cohort studies combined). Six studies were included, no association between number of complications and hand-assistance could be observed (OR 0.99, 95% CI 0.49–1.99, p = 0.98).

Also, the results of the initial trials, were compared to the last group. Subsequently, two meta-analyses changed: the first group of published studies comparing HALDN to LDN showed less complications in favor of HALDN (OR = .45, 95%CI.23-.88, I^2^ = 0%), Fig. 8A and B. Regarding EBL, no significant differences were found in the first group of published studies comparing LESS and LDN (MD = -17.9, 95%CI-26.7- -9.1, I^2^ = 0%) (data not shown).

In Table 6 the severity of complications for each technique modification is shown. Injuries of intraperitoneal organs only occurred in techniques with a transperitoneal approach (*i*.*e*. *LDN*, *HALDN and LESS)*. When all mild complications were excluded from the analysis there were still significantly less complications in the retroperitoneoscopic group as compared to the transperitoneal group (OR = .51, 95CI%. 28-.92, I^2^ = 0%) (data not shown).

**Table 6:** Complications and estimation of severity.

		Incidence of complications (%)				
		LDN (n = 1802)	HALDN (n = 870)	RDN (n = 165)	HARDN (n = 150)	LESS (n = 209)
Intra-operative complications						
Severe	Renal and/or caval vein bleeding	5 (0.3)	5 (0.6)	0 (0.0)	0 (0.0)	0 (0.0)
	Renal artery and/or aortic bleeding	12 (0.7)	3 (0.3)	2 (1.2)	0 (0.0)	0 (0.0)
	Splenic lesion > 500mL blood loss	1 (0.1)	0 (0.0)	0 (0.0)	0 (0.0)	0 (0.0
	Bleeding, undefined > 500 mL blood loss	1 (0.1)	0 (0.0)	0 (0.0)	4 (2.7)	0 (0.0)
	**Subtotal**	**19 (1.1)**	**8 (0.9)**	**2 (1.2)**	**4 (2.7)**	**0 (0.0)**
Moderate	Splenic lesion	14 (0.8)	1 (0.1)	0 (0.0)	0 (0.0)	2 (1.0)
	Bladder lesion	0 (0.0)	0 (0.0)	0 (0.0)	0 (0.0)	1 (0.5)
	Diaphragm lesion	2 (0.1)	0 (0.0)	0 (0.0)	0 (0.0)	1 (0.5)
	Adrenal hematoma	2 (0.1)	2 (0.2)	0 (0.0)	1 (0.7)	0 (0.0)
	Serosa leasion	6 (0.3)	2 (0.2)	0 (0.0)	0 (0.0)	1 (0.5)
	Lumbal vein bleeding	3 (0.2)	1 (0.1)	0 (0.0)	0 (0.0	0 (0.0)
	Adrenal vein bleeding	4 (0.2)	3 (0.3)	1 (0.6)	0 (0.0)	0 (0.0)
	**Subtotal**	**31 (1.7)**	**9 (1.0)**	**1 (0.6)**	**1 (0.7)**	**5 (2.4)**
Mild	Transient CO2 PNP	2 (0.1)	0 (0.0)	0 (0.0)	0 (0.0)	0 (0.0)
	Decapulsation/leasie allograft	1 (0.1)	2 (0.2)	0 (0.0)	0 (0.0)	0 (0.0)
	Other	7 (0.4)	2 (0.2)	0 (0.0)	0 (0.0)	0 (0.0)
	**Subtotal**	**10 (0.6)**	**4 (0.5)**	**0 (0.0)**	**0 (0.0)**	**0 (0.0)**
	**Total**	**60 (3.3)**	**21 (2.4)**	**3 (1.8)**	**5 (3.3)**	**5 (2.4)**
Post-operative complications						
Severe	Pancreatitis	2 (0.1)	2 (0.2)	0 (0.0)	0 (0.0)	0 (0.0)
	Myocardial ischemia	0 (0.0)	0 (0.0)	1 (0.6)	0 (0.0)	0 (0.0)
	Pulmonary embolism	0 (0.0)	1 (0.1)	0 (0.0)	0 (0.0)	0 (0.0)
	Rhabdomyolysis	1 (0.1)	0 (0.0)	0 (0.0)	0 (0.0)	0 (0.0)
	**Subtotal**	**3 (0.2)**	**2 (0.2)**	**1 (0.6)**	**0 (0.0)**	**0 (0.0)**
Moderate	Bowel obstruction	20 (1.1)	12 (1.4)	0 (0.0)	0 (0.0)	4 (1.9)
	Atelectasis	2 (0.1)	0 (0.0)	0 (0.0)	0 (0.0)	0 (0.0)
	Chyleous ascites	5 (0.3)	3 (0.3)	0 (0.0)	0 (0.0)	0 (0.0)
	Chylothorax or pleural effusion	0 (0.0)	0 (0.0)	3 (1.8)	0 (0.0)	0 (0.0)
	Incisional hernia	5 (0.3)	2 (0.2)	0 (0.0)	1 (0.7)	0 (0.0)
	Incisional neurinoma	1 (0.1)	1 (0.1)	0 (0.0)	0 (0.0)	0 (0.0)
	Blood transfusion	26 (1.4)	1 (0.1)	3 (1.8)	0 (0.0)	0 (0.0)
	Urethral obstruction	2 (0.2)	1 (0.1)	1 (0.6)	0 (0.0)	0 (0.0)
	Re-operation for (suspect) bleeding	5 (0.3)	1 (0.1)	1 (0.6)	0 (0.0)	0 (0.0)
	Pneumonia	10 (0.6)	1 (0.1)	2 (1.2)	2 (1.3)	0 (0.0)
	Other	7 (0.4)	1 (0.1)	1 (0.6)	0 (0.0)	1 (0.5)
	**Subtotal**	**83 (4.6)**	**23 (4.6)**	**11 (6.7)**	**3 (2.0)**	**5 (2.4)**
Mild	Urinary retention	6 (0.3)	3 (0.3)	1 (0.6)	0 (0.0)	3 (1.4)
	Wound infection	38 (2.1)	8 (0.9)	3 (1.8)	4 (2.7)	4 (1.9)
	Conjunctivitis, corneal abrasion	0 (0.0)	1 (0.1)	0 (0.0)	0 (0.0)	2 (1.0)
	Scrotal edema	1 (0.1)	2 (0.2)	0 (0.0)	1 (0.7)	0 (0.0)
	Chronic pain (wound, testicular)	4 (0.2)	3 (0.3)	1 (0.6)	0 (0.0)	6 (2.7)
	Subcutaneous hematoma	5 (0.3)	1 (0.1)	0 (0.0)	1 (0.7)	0 (0.0)
	Wound seroma	3 (0.2)	0 (0.0)	0 (0.0)	1 (0.7)	0 (0.0)
	Anemia	3 (0.2)	0 (0.0)	0 (0.0)	0 (0.0)	0 (0.0)
	Urinary tract infection	0 (0.0)	0 (0.0)	0 (0.0)	1 (0.7)	1 (0.5)
	Atypical chest pain	0 (0.0)	0 (0.0)	0 (0.0)	0 (0.0)	2 (1.0)
	Fever	2 (0.1)	1 (0.1)	1 (0.6)	0 (0.0)	2 (1.0)
	Other	2 (0.1)	3 (0.3)	0 (0.0)	0 (0.0)	1 (0.5)
	**Subtotal**	**65 (3.6)**	**22 (2.5)**	**6 (3.6)**	**8 (5.3)**	**21 (10.0)**
	**Total**	**210 (11.7)**	**47 (5.4)**	**18 (10.9)**	**11 (7.3)**	**26 (12.4)**
Not specified*		30 (1.7)	21 (2.4)	0 (0.0)	3 (2.0)	0 (0.0)
	**Total**	**240 (13.3)**	**89 (10.2)**	**21 (12.7)**	**19 (12.7)**	**31 (14.8)**

* Mjoen *et al*.[31]

### Publication bias

**Fig 9:**
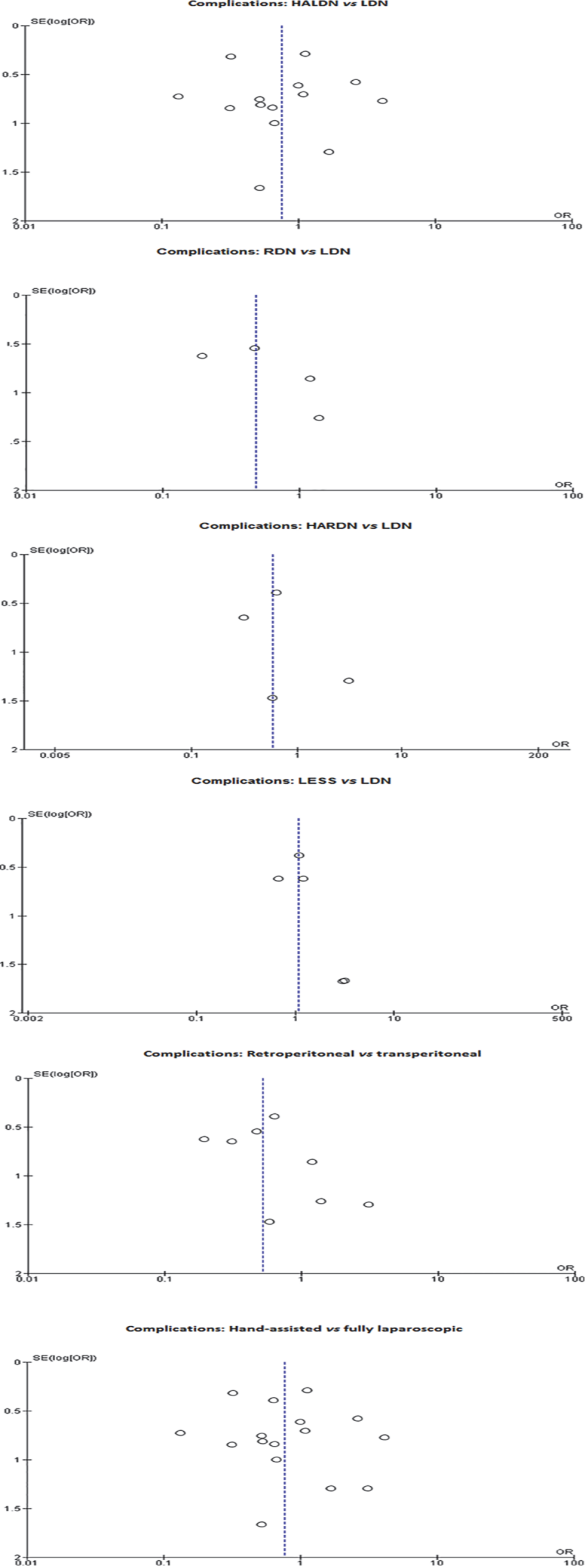
9a: Funnel plot comparing complications in HALDN versus LDN. 9b. Funnel plot comparing complications in RDN versus LDN. 9c: Funnel plot comparing complications in HARDN versus LDN. 9d: Funnel plot comparing complications in LESS versus LDN. 9e: Funnel plot comparing complications in retroperitoneal versus transperitoneal approach. Studies at the bottom tend to cluster towards the right. 9f: Funnel plot comparing complications in hand-assisted versus fully laparoscopic approach.

Funnel plots showed possible publication bias for the comparison of the number of complications in the retroperitoneal *versus* the transperitoneal group (Fig. 9E). The remaining funnel plots were either symmetric or contained insufficient data for meaningful analysis (Fig. 9A, 9B, 9C, 9D and 9F).

## Discussion

This meta-analysis shows that the retroperitoneoscopic approach is associated with less complications as compared to the transperitoneal approach. In fact, when comparing the retroperitoneoscopic approach with LDN, all intra-abdominal injuries occurred in the transperitoneal LDN group.

The data also show that hand-assistance, either through the transperitoneal or retroperitoneal approach, is associated with shorter WIT1 and ORT. Obviously, the association with shorter WIT1 can be explained by the fact that HALDN includes a direct kidney retrieval without the use of an endobag. Nevertheless, there was considerable heterogeneity between the studies regarding WIT1 (I^2^ = 93%). In contrast with the other studies, WIT1 described by Branco *et al*. was remarkably increased in the HALDN group as compared to the standard laparoscopic donor nephrectomy group [24]. Most likely due to a later introduction HALDN, after the initial learning curve with LDN. The association of HALDN with shorter ORT can be explained by the fact that in most studies the surgeons went through their initial learning curve with LDN. However, in three studies, in which ORT was longer for HALDN, no information was provided regarding the learning curve of each separate technique modification.

With regard to the assumption that hand-assistance leads to easier control in case of bleeding, it is remarkable to note that both the HALDN and HARDN technique were not associated with reduced EBL. This may indicate that hand-assistance may not be a major determinant of EBL.

In general, the single port technique is technically more demanding as compared to the three (or four) port approach. Therefore, it is likely to assume that the single port procedures were performed by skilled surgeons, this may explain why LESS was associated with significantly less blood loss. When trials were divided in two equal groups based on year of publication, the earliest studies (2001–2006) comparing HALDN and LDN showed less complications in favor of HALDN [24, 25, 27, 28, 31, 32, 36]. This suggests that HALDN is a safer technique for surgeons of centers in their learning curve.

### Strengths and limitations

The major strengths of this SRMA is the systematic approach with a protocol published in advance and a relatively large number of studies included. A comprehensive assessment is provided of 4 different technique modifications of laparoscopic donor nephrectomy that is relevant to both clinicians and patients. The presented data provide a complete overview of current literature and reveals the gaps in evidence. Several limitations should be discussed. First, the results should be interpreted cautiously, in general, the quality of the included cohort studies and RCTs was low to intermediate or unclear. For 3 randomized clinical trials the risk of bias was high [18, 25, 43], for the remaining 2 trials the risk of bias was unclear [17, 42]. Assessment of the cohort studies revealed that the overall quality was low to intermediate. Second, considerable heterogeneity in most studies was observed. This may be explained by differences in experience and learning curve. In all studies, very limited or no information regarding experience of the surgeons of each separate technique modification was provided. Third, publication bias cannot be excluded, as asymmetry in one funnel plot was found, while most funnel plots contained insufficient data for meaningful evaluation. Fourth, as many studies described their early experience with new technique modifications a certain degree of confounding by indication may have occurred. However, in most studies there were no significant differences in baseline characteristics between the treatment groups including age, gender, BMI, multiple renal arteries and side of nephrectomy. Finally, relevant outcome measures such as postoperative pain and quality of life were not included in the analyses, as these variables were poorly reported.

### Implications for clinical practice

The data show that the retroperitoneoscopic approach is associated with less complications. Furthermore, hand assistance was associated with less complications in the earliest studies. Therefore, it could be suggested that in centers who are converting from open donor nephrectomy to a minimal invasive, laparoscopic approach, safety may increase by the use of the hand-assisted, retroperitoneoscopic (HARDN) technique.

### Conclusion

During laparoscopic kidney retrieval, hand-assistance reduces the operation and first warm ischemia times and may improve safety for surgeons or centers with less experience in laparoscopic donor nephrectomy. The retroperitoneoscopic approach is associated with less complications. However, given the, in general, poor to intermediate quality and considerable heterogeneity in the included studies, further high-quality studies are required.
